# Addressing tuberculosis control in fragile states: Urban DOTS experience in Kabul, Afghanistan, 2009-2015

**DOI:** 10.1371/journal.pone.0178053

**Published:** 2017-05-31

**Authors:** G. Qader, A. Hamim, M. Sayedi, M. Rashidi, L. Manzoor, M. K. Seddiq, N. Ikram, P. G. Suarez

**Affiliations:** 1 Challenge TB Project, Management Sciences for Health, Kabul, Afghanistan; 2 National TB Program, Ministry of Public Health, Kabul, Afghanistan; 3 Office of Health and Nutrition, United States Agency for International Development, Kabul, Afghanistan; 4 Management Sciences for Health, Arlington, Virginia, United States of America; McGill University, CANADA

## Abstract

Tuberculosis (TB) is a major public health problem in Afghanistan, but experience in implementing effective strategies to prevent and control TB in urban areas and conflict zones is limited. This study shares programmatic experience in implementing DOTS in the large city of Kabul. We analyzed data from the 2009–2015 reports of the National TB Program (NTP) for Kabul City and calculated treatment outcomes and progress in case notification using rates, ratios, and confidence interval. Urban DOTS was implemented by the NTP in partnership with United States Agency for International Development (USAID)-funded TB projects, the World Health Organization (WHO), and the private sector. Between 2009 and 2015, the number of DOTS-providing centers in Kabul increased from 22 to 85. In total, 24,619 TB patients were enrolled in TB treatment during this period. The case notification rate for all forms of TB increased from 59 per 100,000 population to 125 per 100,000. The case notification rate per 100,000 population for sputum-smear-positive TB increased from 25 to 33. The treatment success rate for all forms of TB increased from 31% to 67% and from 47% to 77% for sputum-smear-positive TB cases. The treatment success rate for private health facilities increased from 52% in 2010 to 80% in 2015. In 2013, contact screening was introduced, and the TB yield was 723 per 100,000—more than two times higher than the estimated national prevalence of 340 per 100,000. Contact screening contributed to identifying 2,509 child contacts of people with TB, and 76% of those children received isoniazid preventive therapy. The comprehensive urban DOTS program significantly improved service accessibility, TB case finding, and treatment outcomes in Kabul. Public- and private-sector involvement also improved treatment outcomes; however, the treatment success rate remains higher in private health facilities. While the treatment success rate increased significantly, it remains lower than the national average, and more efforts are needed to improve treatment outcomes in Kabul. We recommend that the urban DOTS approach be replicated in other countries and cities in Afghanistan with settings similar to Kabul.

## Introduction

Tuberculosis (TB) is a global public health challenge. In 2015, the largest number of new TB cases was in the Southeast Asia and Western Pacific Regions, accounting for 58% of new cases globally [1]. Rapid urbanization and overcrowded housing in high-density slums contribute to the high TB incidence rates in those regions [2, 3]. The United Nations estimates that about 3.9 billion people live in urban areas, a number that is expected to increase in the less urbanized areas of Asia and Africa [4]. The same trend affects the capital city of Afghanistan. Kabul has experienced a high rate of urbanization caused by internal migration of those seeking jobs or fleeing from conflict. Other studies have shown that the TB burden is high in urban areas but is not related to informal settlements [5]. In Brazil, high TB prevalence rates are reported in dense urban areas but are not necessarily related to income level [6].

Of Afghanistan’s estimated 33 million people approximately 4.4 million live in Kabul City. According to the Ministry of Public Health (MOPH), conflict in Afghanistan has weakened the health care system and led to overcrowded living conditions and a poor quality of life that facilitates TB transmission (Urban DOTS Strategic Plan for five cities [Kabul, Mazar-i-Sharif, Herat, Kandahar, Jalalabad]–Afghanistan, 2015–2019; 2015). The estimated incidence of all forms of TB in Afghanistan is 189 per 100,000 population [1], the prevalence of all forms of TB is 340 per 100,000 [7], and the TB death rate is 37 per 100,000 [1]. Annually, there are an estimated 62,370 cases of all forms of TB in the country, but only 37,001 new TB cases were notified in Afghanistan in 2015. The treatment success rate (TSR) for all forms of new and relapse TB nationwide is 87% [1].

In 2009, only 22 health facilities in Kabul provided partial TB services. As a result, Kabul had poor TB indicators in comparison with national levels. For instance, in 2009 the TB case notification rate was only 59 per 100,000, the TSR for all forms of TB was 31%, and the cure rate for sputum-smear-positive (SS+) TB was 47%. Few private-sector providers had been trained on national TB clinical guidelines, resulting in misdiagnosis, misclassification, improper treatment combinations, and incorrect treatment (MOPH Urban DOTS Strategic Plan 2015).

To address this disparity, the Afghanistan National TB Program (NTP), with assistance from the US Agency from International Development (USAID) and the World Health Organization (WHO), developed an urban DOTS approach in Kabul. This paper describes our experiences in improving DOTS services in Kabul from 2009 to 2015.

## Methods

### The setting

#### Administrative

In 2009, Kabul was home to 3.27 million people. Kabul is the primary referral center for the neighboring 16 districts and is the highest referral point for TB services in the country. In 2015, the total population living in Kabul reached 4.37 million and there were 25 sub-districts.

#### Health system structure

In 2009, there were 137 public and private health facilities of which 106 (77%) provided laboratory services. Twenty-two were public health facilities (16% of the health facilities in Kabul) and provided DOTS. Of those, most provided partial TB services with poor diagnostic and treatment quality and weak infrastructure (MOPH, National Report, 2015). This distribution was not regular. Fourteen of 22 sub-districts were covered, and 54% (12) of DOTS centers were located in four sub-districts; none of the private facilities were covered by DOTS. Per the 2015 National Health Management Information System (HMIS) report, Kabul has 282 public and private health facilities. Of those, 132 (76 public and 56 private) provided laboratory services. DOTS coverage reached 85 (33%) health facilities (69 public and 16 private). Ninety percent of public facilities and 28% of private facilities were covered by DOTS.

The city’s health infrastructure is among the poorest in the country, with 65% of Kabul’s primary health facilities located in rented houses (MOPH, National Surveillance Report, 2009).

### The urban DOTS program approach

The urban DOTS model in Kabul focuses on four major intervention areas: (1) building the capacity of the NTP and health care providers; (2) expanding DOTS coverage in public and private health facilities; (3) improving management and drug supply at health facilities; and (4) improving surveillance, supervision, and monitoring.

In July 2009, the NTP, TB Control Assistance Program (TB CAP) of USAID, and WHO assigned a team to implement and support an urban DOTS program in Kabul City and simultaneously established an urban DOTS taskforce to assist the NTP with developing implementation guidelines and standard operating procedures and creating coordinated and collaborative approaches among sectors including the private sector. The team conducted an assessment, shared the results with senior staff of the MOPH/NTP, private sectors, other non-MOPH sectors, and other stakeholders and partners to obtain political and technical support for the expansion of DOTS in Kabul. During the same year, the NTP presented the urban DOTS program to stakeholders through an orientation workshop. Through this consultative process, the NTP and TB CAP developed an integrated package of standard operating procedures for case detection, TB treatment monitoring for adults and children, TB infection control, community-based DOTS, and surveillance. Following the development of these procedures, from 2009 to 2015, 681 health workers (physicians, laboratory technicians, and nurses) were trained to apply them.

Between 2009 and 2015, the multisectoral urban DOTS program was scaled up to 85 health facilities from various sectors, such as the public and private sectors, ministries of interior, defense, and justice, and the Afghan Red Crescent Society (ARCS). Urban DOTS was implemented in the following phases.

During the scale-up process, the health facilities were assessed to ensure that they met the standards for becoming a DOTS facility The selection criteria were facilities that served large populations, had a high patient volume, covered a diverse population, and were willing to participate voluntarily. The NTP and partners selected health facilities for DOTS implementation and prepared an implementation plan for each facility. The implementation process started and was monitored through routine supervision and monitoring visits. Lastly, the performance of each facility was monitored during quarterly review workshops.

After the selection of health facilities, an action plan was developed and implementation began. Through this process, the MOPH/NTP issued unique identification codes to the selected facilities to provide free TB diagnostic and TB treatment services. At the same time, the MOPH/NTP signed a memorandum of understanding with the private health facilities to delineate the roles and responsibilities in the urban DOTS program.

Public and private health care providers were trained together and the same training curricula, presentations, and standard operating procedures for TB case finding and treatment were applied. Sputum laboratory microscopy was performed in public health facilities and 20 private facilities performed it as well. A similar number of private health facilities were referring presumptive TB patients for diagnosis to public facilities. The NTP provided drugs, supplies, and standard TB recording and reporting forms to private facilities to ensure that they provided quality TB services.

The NTP team was then tasked with ensuring the regular supply of free reagents, anti-TB drugs, and other consumables to the public and private health facilities. The private health facilities were also provided with furniture, and their laboratory rooms and sputum collection points were renovated. The staffs at health facilities selected for DOTS were trained on recording and reporting as well as on the use of data for program improvement. Additionally, selected DOTS health facilities were renovated to enable TB infection control measures.

The diagnostic capacity of the health facilities was strengthened through the implementation of TB microscopy in each facility with laboratory capacity and through the design of a decentralized external quality assurance system. The support included training laboratory personnel, providing standard registers, supplying reagents, and providing quarterly supportive supervision and on-site technical support.

The NTP also introduced the contact investigation strategy in 2013, which was implemented in Kabul under urban DOTS. The household contacts of index cases were screened for symptoms of TB and tested through chest X-ray and sputum examination. The NTP defines an index case as a bacteriologically confirmed pulmonary TB case that results in infection or disease among contacts. The households of index cases defined as close contacts (a person living in the same household with the index case [e.g., the caregiver of the child] or in frequent contact with the index case) are verbally screened for the signs and symptoms of TB. If symptomatic, they are tested for TB (sputum examination and chest X-ray) and the health care workers apply standard procedures for TB case finding and treatment.

**Fig 1:**
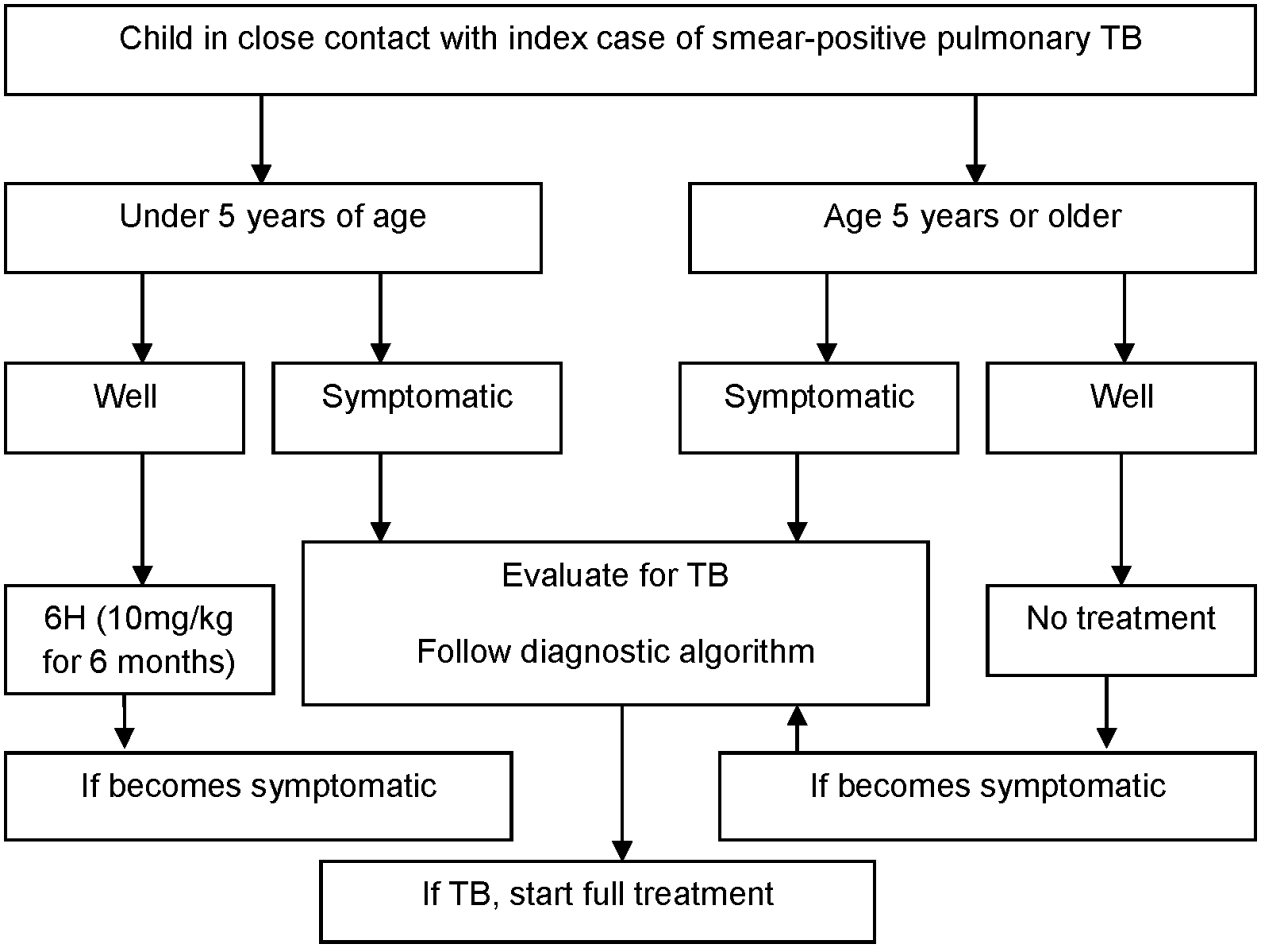
Algorithm for screening children in contact with an index case.

Children under the age of five were screened with chest X-ray and the Mantoux test to diagnose TB. Children who were symptom-free and were not diagnosed with TB received preventive therapy with isoniazid 10 mg/kg (administered daily for six months to prevent the risk of developing TB illness) (NTP, Guidelines for National TB Control, 2015). The health workers conduct symptomatic screening of all family members for TB signs and symptoms with the following actions: They identify the children under the age of five, both sick and healthy. If the children are not symptomatic, they apply isoniazid preventive therapy for six months. If they are symptomatic, the standard operating procedure for TB case finding among children is applied (Fig 1).

The NTP also instituted a quarterly monitoring mechanism to monitor the stock of drugs and reagents at the facility and national levels. Regular supervision and monitoring visits were conducted to verify the clinical quality of diagnosis and treatment. The facilities’ performance was compared to pre-set targets and reviewed during quarterly workshops. During the workshops, the facilities received feedback on their performance, and recommendations were followed up through regular visits to health facilities.

Finally, after a performance review of facilities covered by DOTS, they were moved to the next step of development. As per guidelines for TB control in Afghanistan and standard operating procedure for TB diagnosis and treatment, TB patients are followed by either health care providers or treatment supporters from the community. This includes ensuring follow-up examination at the end of month two or three and month five and the end of treatment and contact with TB patients who are in isoniazid preventive therapy to promote adherence to treatment. Also, if a TB patient moves to another city or facility during treatment, the referring facility reports the treatment outcomes to the refer-out facility.

The MOPH provided overall leadership of health service delivery in DOTS implementation in Kabul and authorized the public and private facilities to implement DOTS by signing a memorandum of understanding with each of the facilities, registering each facility with the HMIS, and issuing a unique code. The NTP’s role was to ensure that these facilities implement the national TB guidelines and standard operating procedures for TB case finding and treatment. The NTP also ensured the supply of drugs, reagents, and other consumable to these facilities through the provision of supportive supervision and monitoring of TB activities. The USAID-funded TB projects provided technical assistance to the NTP and public and private facilities to ensure that activities are implemented as planned and that the facilities had sufficient technical and diagnostic capacity. The role of the private and public sectors was to provide free TB services and apply the NTP standards and guidelines for diagnosis and treatment, contact investigation, TB infection control, and the TB information system, procure manuals, and identify and refer drug-resistant TB cases to the NTP for further investigation.

### Data collection and analysis

The data used in this analysis were collected from health facilities by using standard NTP recording and reporting forms. Frequencies, proportions, ratios, rates, and 95% confidence intervals were used to describe treatment outcomes and case notifications. Case notification rates (CNRs) were computed using the projected population for each year. Given that Afghanistan has very low HIV prevalence, HIV patients were not included in our calculations.

### Ethics statement

We used routine programmatic data for this analysis and thus no ethical approval was sought. The urban DOTS implementation was planned and implemented with the leadership of the NTP. The NTP reviewed and approved the manuscript for publication in a peer-reviewed journal.

## Results

### Building the capacity of health workers and expanding DOTS

Using the new NTP guidelines and standard operating procedures, 681 physicians, laboratory technicians, and nurses from health centers and private and public hospitals were trained on DOTS. Between 2009 and the end of 2015, the number of DOTS health facilities increased from 22 to 85 facilities owned by the MOPH, other governmental agencies, the private sector, and the Afghan Red Crescent Society. The initiation of DOTS in 85 health facilities in Kabul decreased the population covered by each DOTS facility from 1 facility per 148,636 population in 2009 to 1 per 51,411 population in 2015. A TB patient referral system from community volunteers and lower-level health facilities to the DOTS centers was established. The lower-level health facilities identify presumptive TB cases and refer them to upper-level health facilities (diagnostic centers). After diagnosis, people with TB are referred back to their locality to begin treatment. Another accomplishment between 2009 and 2015 was an increased number of laboratories providing microscopy services, from 106 to 132 (Table 1). Finally, all visitors to health facility outpatient departments were screened for TB using the NTP’s standard operating procedures for case detection.

**Table 1:** Contribution of urban DOTS to TB service delivery in Kabul city, 2009–2015.

	2009 N (%)	2010 N (%)	2011 N (%)	2012 N (%)	2013 N (%)	2014 N (%)	2015 N (%)
**Characteristics**							
***DOTS coverage***							
Total population, Kabul	3.27 M	3.57 M	3.82 M	3.95 M	4.09 M	4.23 M	4.37 M
No. of health facilities	137	145	182	189	192	216	282
Facilities with lab services	106 (77)	111 (77)	111 (61)	112 (59)	120 (63)	131 (61)	132 (47)
Health facilities providing DOTS	22 (16)	48 (33)	53 (29)	68 (36)	73 (38)	80 (37)	85 (30)
No. of private health facilities	NA	40	49	49	50	53	56
Private health facilities providing DOTS	NA	10 (25)	14 (29)	17 (35)	21 (42)	22 (42)	26 (46)
Population per DOTS facility	148,636	74,375	72,075	58,088	56,027	52,875	51,411
***Case notification***							
Outpatient attendance	232,367	340,394	374,866	412,352	474,205	545,536	627,136
Presumptive TB patients identified/ examined	2,856 (0.13)	10,150 (3)	11,900 (3)	13,644 (3.3)	14,181 (3)	17,061 (3)	17,525 (3)
TB cases notified (all forms)	1,934 (31) (SD = 38.7, CI = 16.2)	2,738 (41)	2,728 (38)	3,215 (43)	3,548 (46)	5,007 (63)	5,449 (66) (SD = 23.4, CI = 4.9)
New SS+ TB cases notified	871 (32) (SD = 16.1, CI = 6.7)	1,022 (36)	1,082 (36)	1,174 (38)	1,204 (37)	1,280 (38)	1,449 (42) (SD = 9, CI = 1.9)
Case notification rate per 100,000 population (all forms)	59	77	71	81	87	118	125
Case notification rate per 100,000 population (SS+)	25	29	28	30	29	30	33
***Treatment outcomes (all forms TB)***							
Treatment success rate	601 (31)	1,305 (48)	1,695 (62)	2,069 (64)	2,369 (67)	3,186 (64)	3,651 (67)
Not evaluated	1,206 (62)	1,184 (62)	791 (29)	889 (28)	893 (25)	1,418 (28)	1,308 (24)
Lost to follow-up	86 (5)	158 (12)	166 (6)	169 (5)	194 (5)	313 (6)	327 (6)
Treatment failed	7	38 (1)	14 (1)	30 (1)	25 (1)	23 (1)	54 (1)
Died	34 (2)	53 (2)	62 (2)	58 (2)	67 (2)	67 (1)	109 (2)
***Treatment outcomes (new SS+ TB)***							
New SS+ TB started on treatment	814 (94)	1,022 (100)	1,082 (100)	1,174 (100)	1,204 (100)	1,280 (100)	1,449 (100)
Cured	382 (47)	531 (52)	682 (63)	740 (63)	783 (65)	845 (66)	985 (68)
Treatment success rate	382 (47)	634 (62)	736 (68)	821 (70)	879 (73)	947 (74)	1,116 (77)
Not evaluated	358 (44)	297 (29)	248 (23)	258 (22)	229 (19)	230 (18)	233 (16)
Lost to follow-up	50 (6)	61 (6)	65 (6)	59 (5)	60 (5)	77 (6)	72 (5)
Treatment failed	8(1)	10 (1)	11 (1)	12 (1)	12 (1)	13 (1)	14 (1)
Died	16 (2)	20 (2)	22 (2)	24 (2)	24 (2)	13 (2)	14 (1)
***TB cases diagnosed among children* < 15**	180 (9)	235 (9)	198 (7)	238 (7)	383 (10)	1,317 (26)	1,080 (120)
***Contact investigation***							
Index cases (BC) investigated	NA	NA	NA	399	571	809	1,208
Contacts (symptomatic) screened for TB	NA	NA	NA	1,994	2,855	4,046	6,040
Contacts presumed to have TB	NA	NA	NA	195 (10)	219 (8)	318 (8)	645 (11)
TB cases notified among contacts	NA	NA	NA	18 (1)	14 (0.5)	23 (0.6)	53 (1)
Children < 5 among contacts	NA	NA	NA	415 (42)	580 (100)	678 (84)	836 (69)
Children < 5 put on IPT	NA	NA	NA	119 (29)	495 (85)	519 (77)	767 (92)

SD, standard deviation; CI = confidence interval.

### Case notification rate of new TB cases and treatment outcomes

**Fig 2:**
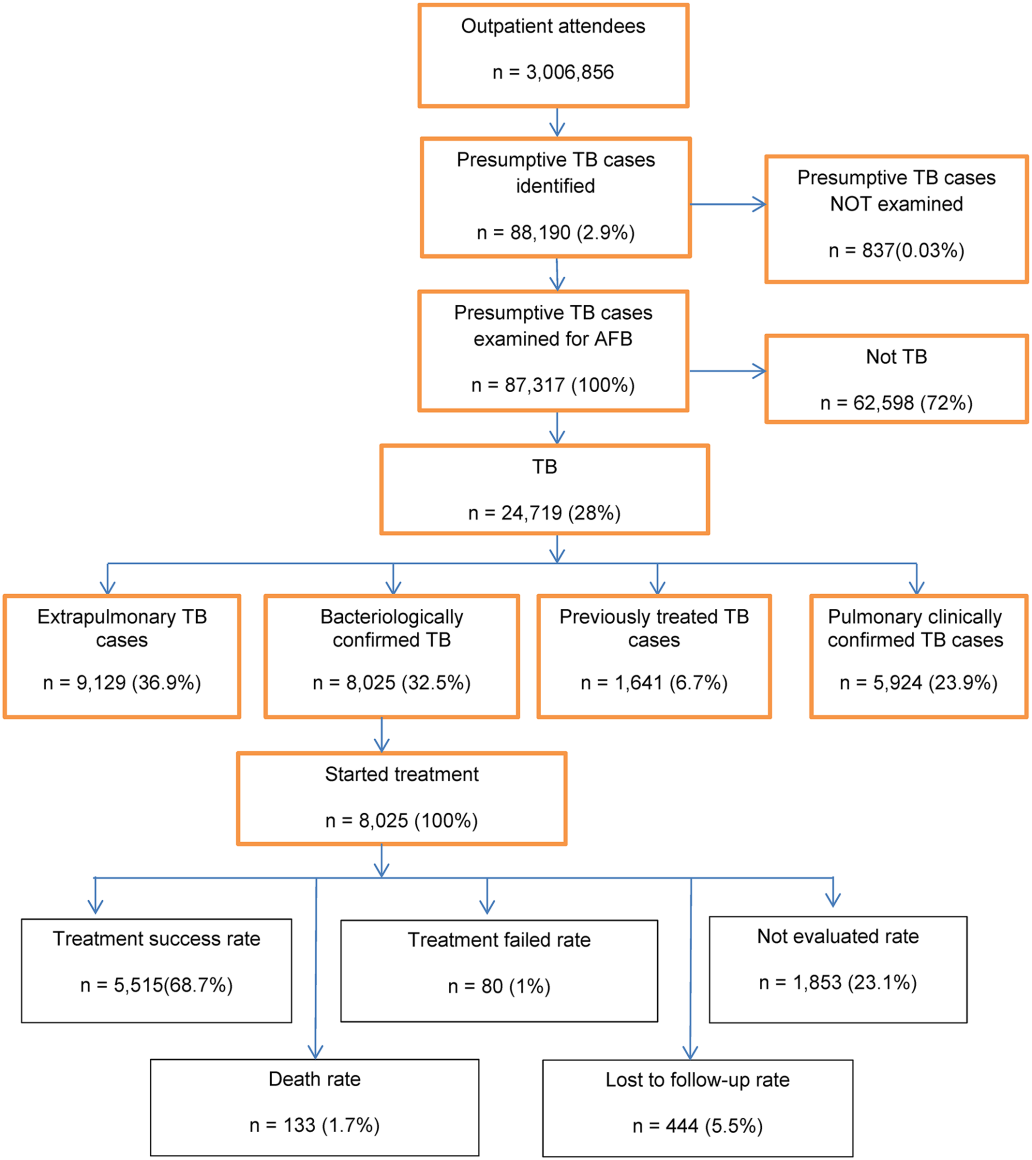
Flow chart of TB case notification and treatment outcomes in Kabul, 2009–2015.

**Fig 3:**
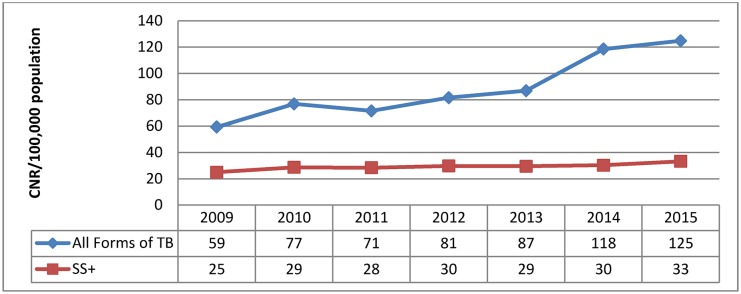
Case notification rate per 100,000 population in Kabul, 2009–2015.

A total of 24,619 of all forms of TB cases were detected in Kabul from 2009 to 2015. Among the detected cases, 8,025 (32.4%) were SS+ TB (Fig 2). The CNR for all forms of TB improved from 59 cases per 100,000 population to 125 cases per 100,000 population between 2009 and 2015 respectively. The CNR for SS+ TB also increased from 25 cases per 100,000 population to 33 cases per 100,000 population for the same period (Fig 3).

**Fig 4:**
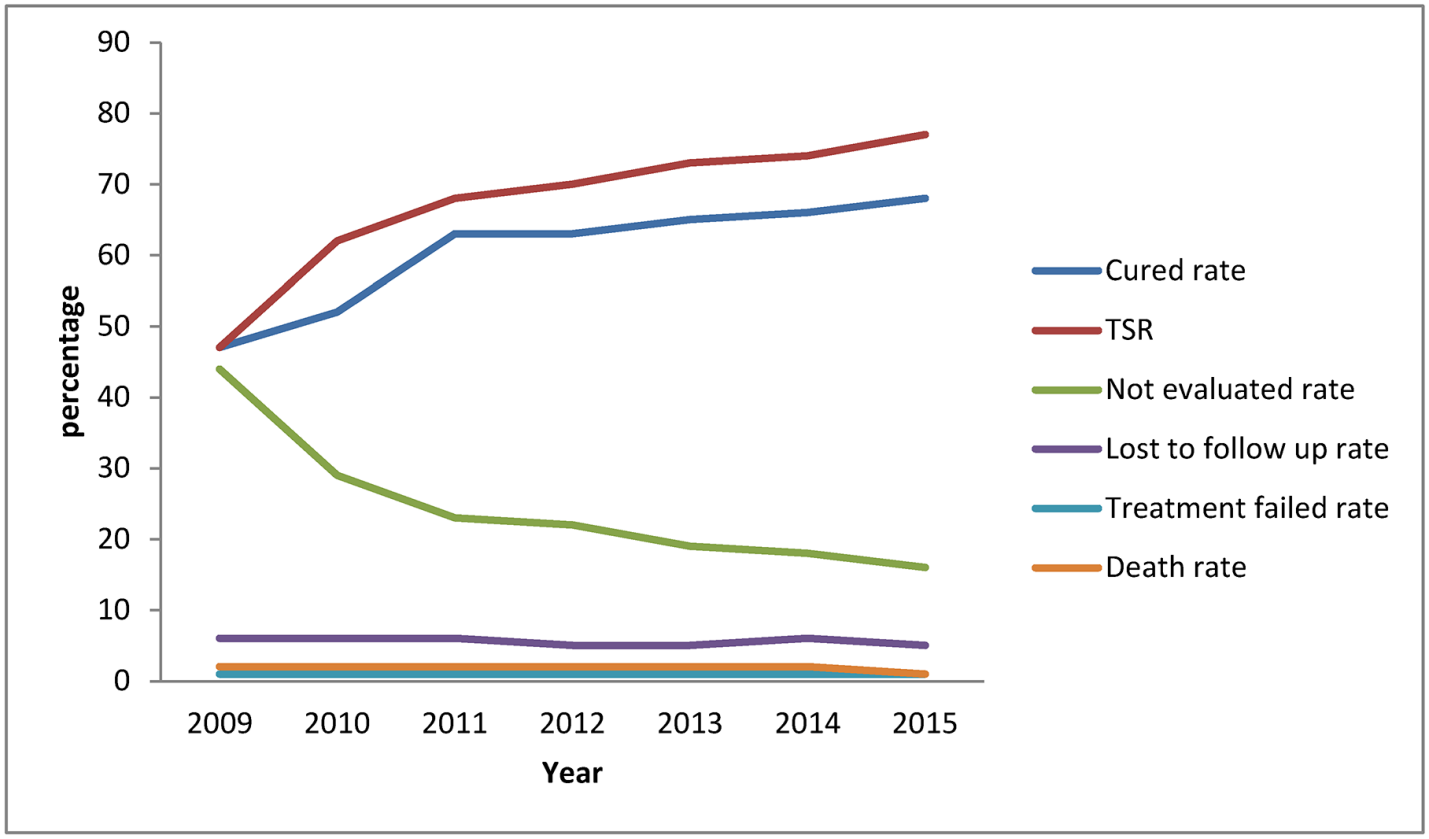
Treatment outcomes for SS+ TB, Kabul, 2009–2015.

The TSR for all forms of TB increased from 31% in 2009 to 67% in 2015. Likewise, the TSR for SS+ TB cases improved from 47% in 2009 to 77% by the end of 2015. The SS+ TB cure rate also improved, from 47% to 68%. The not-evaluated rate for 2009–2015 remained high, but it decreased from 62% to 24%. The not-evaluated rate for SS+ TB decreased from 44% in 2009 to 16% in 2015. During the seven-year period, the death rate was constant at 2%, and the lost-to-follow-up rate was consistently between 4% and 6%. The treatment failure rate remained low (1%) (Table 1, Fig 4).

In 2009 there were no reports of TB diagnosis and treatment in private health facilities. In 2010, among the 40 private health facilities, 10 (25%) were willing to start DOTS. By 2015, 26 (46%) of the 56 private health facilities were implementing a full DOTS program. Among the diagnosed cases of all forms of TB during the seven-year period, 1,797 (7.2%) came from the private sector. Interestingly, the TSR for all forms of TB was better in private health facilities—at 52% at 2010, 89% in 2014, and 80% in 2015—as compared to 67% in public health facilities in 2015 (Tables 1 and 2).

**Table 2:** Treatment outcomes in private facilities, 2009–2015.

	2009 N (%)	2010 N (%)	2011 N (%)	2012 N (%)	2013 N (%)	2014 N (%)	2015 N (%)
**Characteristics**	0						
All forms TB cases notified	NA	120	182	267	213	410	605
Treatment success rate	NA	63 (52)	166 (91)	175 (66)	147 (69)	363 (89)	482 (80)
Not evaluated rate	NA	31 (26)	1 (0.5)	69 (26)	38 (18)	0 (0)	21 (4)
Lost to follow-up rate	NA	2 (1.5)	9 (5)	9 (3)	7 (3)	29 (7)	48 (8)
Treatment failure rate	NA	1 (1)	0 (0)	1 (0.5)	1 (0.5)	0 (0)	0 (0)
Death rate	NA	2 (1.5)	1 (0.5)	5 (2)	1 (1)	10 (2.5)	16 (3)

#### Contact screening

Contact screening of SS+ TB index cases was introduced in the urban TB program in 2013. A total of 14,935 contacts were screened for TB, and 1,377 (9.2%) had presumptive TB. Of these presumptive TB cases, 108 (7.8%) were diagnosed with TB. This makes the yield of TB among contacts 723 per 100,000 population. The private sector registered 707 household contacts and identified 38 as presumptive TB; of those, 37 were tested for TB and 2 new SS+ TB cases were diagnosed. The private sector’s overall contribution to contact investigation is 5% for households registered and 2% in TB case notification among contacts screened for TB during 2013–2015.

#### Childhood TB

A total of 2,509 (67%) children under the age of five were registered as contacts and 2,004 (80%) were screened for TB. Of those found to be negative for TB, 1,900 (76%) were put on isoniazid preventive therapy (Table 1).

A total of 3,631 children under the age of 15 were notified in seven years. The number of children diagnosed as compared to the rest of the age groups was 180 (9.3%) in 2009; in 2015 the number increased to 1,080 (19.4%) (Table 1).

## Discussion

The data from Kabul provide operational and epidemiological evidence that implementation of the urban DOTS program between 2009 and 2015 resulted in significant improvements in TB service expansion, case finding, and treatment outcomes in a country experiencing ongoing conflict. The data show that the NTP and partners such as USAID, Japanese International Cooperation Agency, Global Fund to Fight AIDS, Tuberculosis and Malaria, and WHO were able to more than double the number of DOTS-providing health facilities in Afghanistan, leading to an improvement (decline) in the population-to-DOTS facility ratio of more than 50%. At the same time, the CNR for all forms of TB increased from 59 cases per 100,000 population in 2009 to 125 cases per 100,000 population in 2015, and the number of SS+ TB cases per 100,000 population rose from 25 to 33. The program also led to significant improvement in the CNR for both all forms of TB and SS+ TB. Improvements can be attributed to integrated urban community-based TB screening, health facility–level screening of all outpatient department visitors, contact screening strategies, and better diagnostic capacity because of strengthening of the TB laboratory network in Kabul.

It should also be noted that many patients from adjacent provinces and throughout the country prefer to travel to Kabul to see more qualified health professionals and receive better diagnostic services. It is evident from the data that the transfer-out rate within the not-evaluated rate is very high even though it gradually decreased because patients were referred back to their localities before they started treatment. Regardless, the number of patients travelling to Kabul was high and this may have inflated the CNR (since the denominator to calculate the CNR is only the Kabul population).

The significant increase in the number of pediatric TB cases from 383 cases in 2013 to 1,317 in 2014 (a threefold increase in one year) could be attributed to the implementation of a project for pediatric TB in select children’s hospitals in Kabul. Additional analysis is required to confirm if the data reflect the real pediatric TB situation in Kabul.

Per the 2015 WHO estimates, TB prevalence in Afghanistan was 340 cases per 100,000 population [7] and if we assume the urban prevalence is the same as that of the national level, more than half of the TB patients in Afghanistan are missed. Interventions to increase case detection should be prioritized to address the large number of estimated missed TB cases every year. Case detection should be prioritized through a multi-level approach, starting with increasing strategic investments for TB control; scaling up the use of new technology (digital X-ray machines and GeneXpert), with an emphasis on TB hot spots and high-risk populations (contacts, children, prisoners, health workers, diabetics, and people in congregate settings and slums); and addressing barriers to health-seeking behaviors within the community. The country currently relies heavily on smear microscopy. This approach limits the capacity to diagnose smear-negative patients and multidrug-resistant TB cases.

Another significant achievement of the urban DOTS program in Kabul is an increase in the TSR for all forms of TB from 31% in 2009 to 67% in 2015. In 2015 the national TSR was 87% [1], a rate that is higher than that of Kabul City. The improved TSR resulted from increased access to TB services, free treatment, and improvements in the delivery of directly observed treatment (DOT) services provided by nursing staff in DOT-specific areas in health facilities, with the involvement of patients’ families. The TSR in Kabul remains lower than the national rate due to the large number of patients not evaluated. In reality, many of these patients could have transferred out of the health facility, returned to their homes (outside the city) and continued treatment elsewhere. The TSR also reflects the 2015 death rate (2%), which is higher than the 37 deaths per 100,000 population reported by the WHO [1]. Since the TB services and medical professionals are better in Kabul, many of the patients who come to receive services are in a critical state. Death rates among critical patients are higher than those of patients diagnosed and treated at early stages of the disease. This fact might have inflated the TB death rate in Kabul City.

In major urban centers in Asia and Africa, different TSRs have been reported. In the urban DOTS expansion in Abia State of Nigeria, the TSR for TB patients in slum areas was 88.5% [8]. It was 88% in Latvia [9] and reported to be more than 90% in Nepal [10]. In another study in urban Pakistan and one in Thailand, TSRs of 64% and 62%, respectively, have been reported [11, 12]. Loss to follow-up is a major challenge in urban areas because many patients start their treatment in urban areas but return to their home areas to continue treatment. In our patients the lost-to-follow-up rate was 5–6% during the seven years, which is lower than the rates reported in Kolkata, India (9.4%) [13] and in a slum area of Nairobi, Kenya (13%) [14].

The proportion of patients not evaluated for all forms of TB decreased from 62% to 24%, while the decrease in the proportion of patients not evaluated for SS+ TB dropped from 44% to 16% between 2009 and 2015. The proportion of patients not evaluated is still high and is the main cause of the lowered treatment success and cure rates. Zhuben et al. reported a higher transfer-out rate of 52.6% in selected health facilities in Kabul [15]. In a different setting in Ethiopia, the transfer-out rate declined from 4% to 1% over the course of five years [16]. The situation in Kabul is different from the other country situations described above because progress has been made in a country experiencing conflict and a high influx of internally displaced people, with the health care system functioning suboptimally.

The yield of TB among contacts was 1%, which is lower than the 2.3% reported in Ethiopia [17]. Two systematic reviews reported 4.5% and 3.1% TB yields in low-income and middle-income countries, respectively [18, 19]. In one study in Pakistan, the yield of TB among contacts was 22.7% [20], which is the highest rate reported.

The introduction of TB index contact screening has also contributed to pediatric TB diagnosis, treatment, and prevention. Contact screening, which was nonexistent at baseline, became fully operational during the project period, leading to the screening of 2,509 children under the age of five, with 67% of them receiving isoniazid preventive therapy. The high yield of TB among contacts in Kabul illustrates the magnitude of the TB epidemic within the general population and within the families of index cases in Kabul. The high yield of TB among contacts further demonstrates the importance of scaling up and sustaining the urban DOTS program.

Our study has several limitations. There has been no TB prevalence survey in Afghanistan that could provide accurate estimates of the burden of TB in the country. For this paper the estimates of the TB burden in Kabul were based on WHO estimations and do not accurately reflect the burden of the disease in the country. The level of uncertainty was great; therefore, an accurate estimate of the burden of TB is required for future studies. Although data from the NTP lack the deductive power required for a formal experiment, they do cover a large population and provide information about numerous indicators. There was no disaggregated data about some of the high-risk populations (e.g., prisoners, nomads), given the operational limitations to access to information about these groups.

Although Afghanistan is affected by continuing conflict, the positive results highlighted in this paper demonstrate the importance of NTP-led urban DOTS interventions supported by multiple stakeholders in improving access to and quality of TB services in a challenging environment. The lead role of the NTP in providing robust technical assistance through externally supported mechanisms, and the engagement of broader health-sector components—including other government agencies and the private sector—have been essential success factors. Continued efforts are needed to further improve TB indicators and sustain the achievements of the urban DOTS program in Kabul.

In addition, implementation of the public-private mix has contributed 7.2% of patients notified among all TB cases in Kabul. The urban DOTS program covered private health facilities, whose coverage reached 46% in 2015. Similarly, notified TB cases in the private sector increased from 120 in 2010 to 605 in 2015. The TSR increased from 52% in 2010 to 80% in 2015 and the not-evaluated rate decreased from 26% in 2010 to 4% in 2015. The results in private health facilities are better than in public health facilities. Intrinsic and extrinsic factors motivate the private sector to perform well. Intrinsic motivators could include managing a positive relationship with and receiving recognition from the MOPH, as well as the private sector’s ability to attract and retain clients because of the broader (and better-quality) services the private sector provides. Staff in private facilities generally counsel patients better and are sensitive to their needs. In the public health facilities there are no personal gains related to the quality of services. Another reason might be that more educated and wealthier patients are more likely to choose private-sector services. Private health facilities may cater to more Kabul residents because they are more likely to be able to afford private services. The poor who come from the rural areas may have to resort to public health facilities. Extrinsic motivating factors could include support for renovations; trainings; and free reagents and drugs. In Nigeria, however, the contributions of private for-profit health facilities to TB care were lower than those of public facilities [21]. The major reasons for the difference in contributions were that private practitioners are not trained on the national TB guidelines, which was the case in Kabul in 2009 when we started the urban DOTS program. Similar treatment outcomes were observed in private health facilities in Pakistan, where 84% of patients completed treatment [22].

Furthermore, evidence from Dhaka, Bangladesh, suggests that engagement of private practitioners and institutions such as clinics and hospitals contributed to 36% of all forms of TB case finding [23]. The participatory approach used in the current implementation model appears to have made it possible to promote better coordination and collaboration between the public and private health sectors in Kabul. This has led to the private sector applying public health approaches to identify and diagnose TB cases and refer them to health facilities for treatment and follow-up examinations. Furthermore, the private sector has understood the importance of recording and reporting health events and started implementing a TB surveillance system as a routine practice. It also assisted the NTP to use the private sector’s significant opportunity to identify and diagnose TB according to NTP and MOPH strategies and guidelines.

## Conclusions

Implementation of the urban DOTS model in the densely populated city of Kabul contributed to the institutionalization of TB service delivery within the public and private health sectors. Health facilities in these sectors provided sustainable TB care to their clients, which ultimately improved access to high-quality free TB services. Furthermore, it led to improvements in TB case notification and the TSR in a challenging setting. Based on these successes, it is strongly recommended that DOTS implementation be expanded to other countries and cities in Afghanistan with conditions similar to those of Kabul. The yield of contact screening is also high, and we recommend that it be implemented nationwide. More effort is needed to further improve the treatment success and cure rates in Kabul, which remain lower than the national averages.

## Supporting information

S1 File Case notification Afghanistan 2006–2016. (RAR)
